# Nucleotide Catabolism on the Surface of Aortic Valve Xenografts; Effects of Different Decellularization Strategies

**DOI:** 10.1007/s12265-016-9672-6

**Published:** 2016-02-01

**Authors:** Barbara Kutryb-Zajac, Ada H. Y. Yuen, Zain Khalpey, Paulina Zukowska, Ewa M. Slominska, Patricia M. Taylor, Steven Goldstein, Albert E. Heacox, Marialuisa Lavitrano, Adrian H. Chester, Magdi H. Yacoub, Ryszard T. Smolenski

**Affiliations:** Department of Biochemistry, Medical University of Gdansk, Gdańsk, Poland; Heart Science Centre, Imperial College London, London, UK; University of Arizona, Tucson, AZ USA; CryoLife, Inc, Kennesaw, GA USA; University Milano-Bicocca, Milan, Italy

**Keywords:** (Max 10) heart valves, Xenografts, Nucleotide metabolism, ATP, Adenosine, Valve prostheses

## Abstract

Extracellular nucleotide metabolism controls thrombosis and inflammation and may affect degeneration and calcification of aortic valve prostheses. We evaluated the effect of different decellularization strategies on enzyme activities involved in extracellular nucleotide metabolism. Porcine valves were tested intact or decellularized either by detergent treatment or hypotonic lysis and nuclease digestion. The rates of ATP hydrolysis, AMP hydrolysis, and adenosine deamination were estimated by incubation of aorta or valve leaflet sections with substrates followed by HPLC analysis. We demonstrated relatively high activities of ecto-enzymes on porcine valve as compared to the aortic wall. Hypotonic lysis/nuclease digestion preserved >80 % of ATP and AMP hydrolytic activity but reduced adenosine deamination to <10 %. Detergent decellularization completely removed (<5 %) all these activities. These results demonstrate high intensity of extracellular nucleotide metabolism on valve surface and indicate that various valve decellularization techniques differently affect ecto-enzyme activities that could be important in the development of improved valve prostheses.

## Introduction

Calcific aortic valve disease (CAVD) is a progressive disorder that increasingly afflicts the aging population. This disease appears as a result of thickening and calcification of the aortic valve leaflets and could have form of aortic sclerosis (in the absence of obstruction in blood flow at the valve level) or aortic stenosis (with obstruction to ventricular outflow) [1]. Left ventricular hypertrophy caused by severe aortic stenosis is associated with sudden death, congestive heart failure, and stroke [2]. Aortic sclerosis is evident in more than 25 % of patients over age 65, whereas aortic stenosis occurs in 2 to 5 % of the eldest patients, and it is the second most common indication for cardiac surgery [3, 4]. A surgical approach is the method of choice for treatment for end-stage valvular diseases. Aortic valve replacement (AVR) and transcatheter aortic valve implantation (TAVI) have become routine procedures with the number rising considerably over the last years [5, 6].

Mechanical or bioprosthetic valves are two general options that exist for valve replacement procedures. Mechanical valves display good structural durability but they are susceptible to infection and are associated with the risk of thromboembolic complications. For these patients, lifelong anticoagulation therapy is required, associated with a high risk of spontaneous bleeding and thromboembolism [7]. In the field of biological grafts, cadaveric valves taken from human donors (homografts) eliminate the need of anticoagulation and have the best hemodynamic properties, but their availability is limited. Other mammalian valves (xenografts), such as porcine aortic valves, are readily accessible and the risk of thromboembolic complications is minimal. However, xenografts have to be processed in order to achieve sterility, alter immunogenicity, and enhance their durability through alteration of their physical properties [8]. Decellularization of xenogenic material has become routine procedure to remove antigens and nucleic acids while preserving extracellular matrix (ECM) that provides a biomechanically efficient scaffold [9]. The most common decellularization protocols include steps, such as lysis of the cell membrane by hypotonic/hypertonic treatment or detergent-based processing, followed by enzymatic digestion, and removal of cellular debris by isotonic washout [10–14]. To reduce potential transmission of microbiological hazards, valves are sterilized by ɣ-irradiation since glutaraldehyde sterilization has been associated with scaffold destruction [15]. Despite optimal hemodynamics and resistance to infection, the major problem of bioprosthetic valve durability is progressive degeneration due to chronic inflammation and calcification [16, 17].

Our previous studies identified extracellular adenosine triphosphate (ATP) as a potent regulator of osteoblast differentiation in human valve interstitial cells [18]. Also, other authors reported that ATP and activity of valvular ectonucleotidases engaged in its metabolism could play a substantial role in the development and progression of CAVD [19]. Moreover, extracellular ATP exerts pro-inflammatory and pro-aggregatory actions that promote pathological processes within valves and vessel wall [20, 21]. In addition to ATP (which acts through P2 receptors), its breakdown product—adenosine, also displays many regulatory functions via P1 receptors, widely distributed in the cardiovascular system. The spectrum of extracellular adenosine activities is mostly protective against inflammation, foam cells accumulation, or platelets aggregation—disorders that initiate aortic sclerosis and atherosclerosis [22–24]. Moreover, adenosine is also recognized as a regulator of calcification within the cardiovascular system, since lack of the main adenosine-producing enzyme in vasculature is manifested by arterial calcification [25]. This statement is supported by our previous results indicating that adenosine protected valvular interstitial cells (VICs) against osteoblast differentiation [18]. However, the role of adenosine and enzymes responsible for its metabolism in CAVD still raises controversy [26]. Nonetheless, our studies showed that native intact aortic valves are rich in membrane-bound enzymes that catalyze metabolism of extracellular ATP to adenosine, what is essential in maintaining the balance between these regulatory molecules [27]. The most important role in extracellular nucleotide cascade, on the surface of heart valves, is played by ecto-nucleoside triphosphate diphosphohydrolase 1 (eNTPD-1/CD39), which hydrolyzes ATP to adenosine monophosphate (AMP) through adenosine diphosphate (ADP), ecto-5′-nucleotidase (e5′NT/CD73), which degrades AMP to adenosine and adenosine deaminase (eADA), which is responsible for extracellular adenosine deamination [28, 29].

This study compared the activities of enzymes involved in extracellular nucleotide metabolism in the valve leaflets and aortic wall and evaluated how different methods used for decellularization of xenograft valves affect these activities.

## Methods

### Valve Preparation

Fresh porcine aortic valves (*n* = 8) and ascending aorta fragments (*n* = 4) were obtained from 1-year-old pig hearts from a local slaughterhouse, placed into ice-cold physiologic salt solution and transported to the laboratory on ice within 1 h of harvest. Aortic valve leaflets were dissected from the aortic valve conduit and divided into 0.2-cm^2^ sections. Similar sections were obtained from aortas. These sections were washed in Hank’s Balanced Salt Solution (HBSS) and used in experiments to study native ecto-enzyme activities or used for processing. The first processing method was carried out by hypotonic lysis in deionized water, followed by DNAseI/RNAseA digestion, as previously described [30]. Subsequently, valves were washed, cryopreserved in 10 % DMSO/10 % fetal bovine serum in DMEM and subjected to sterilization by 25–40 kGy of γ-radiation in dry ice. A second processing method applied previously described decellularization procedure based on incubation of valves with 0.1 % sodium dodecyl sulfate (SDS) with 0.1 % EDTA in 10 mM Tris–HCl, pH 8.0 (48 h), followed by nuclease digestion, cryopreservation in DMSO, and sterilization using γ-radiation [31–33]. Prior to analysis, valves were warmed, thawed by immersing the valve package in 40 °C water, and DMSO was washed out by rinsing in phosphate-buffered saline (PBS).

### Analysis of Extracellular Nucleotide Catabolism Enzymes

Intact aorta fragments, native, and processed aortic valve leaflets were incubated in 1 ml of HBSS with substrates for extracellular enzymes. Adenosine, ATP, and AMP at final concentration of 50 μM were sequentially added to 1 ml of HBSS with medium exchange after each substrate. An inhibitor of adenosine deaminase-erythro-9-(2-hydroxy-3-nonyl) adenine (EHNA) was present at 5 μM concentration during incubation with ATP and AMP to block conversion of adenosine to inosine. To ensure that evaluated activities originate exclusively from the action of extracellular enzymes, part of the experiments were conducted with the addition of the nucleoside transport inhibitor: S-(4-Nitrobenzyl)-6-thioinosine (NBTI). Samples were collected after 0, 5, 15, and 30 min of incubation at 37 °C and then directly injected onto HPLC. Concentrations of nucleotides and nucleosides were measured by reversed-phase HPLC according to the method described earlier [34]. The rates of ATP hydrolysis, AMP hydrolysis, and adenosine deamination were calculated from linear phase of the reaction and normalized to the surface area.

### Statistical Methods

Values are presented as mean ± SEM. Statistical analysis was performed using paired Student’s *t* test to compare two groups or one-way analysis of variance (ANOVA) followed by Tukey’s multiple comparison test to compare more than two groups (InStat software, GraphPad, San Diego). A *p* value <0.05 was considered as significant difference.

## Results

**Fig. 1:**
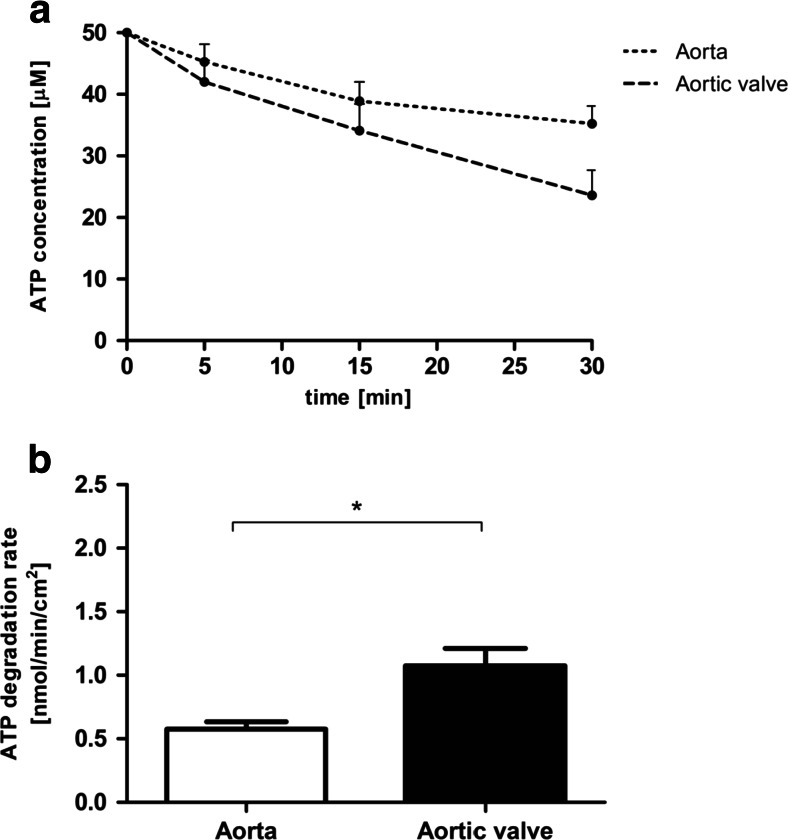
Adenosine triphosphate (ATP) degrading capacity is increased on the aortic valve surface as compared to the vessel surface. ATP concentration during test (**a**) and calculated degradation rate of ATP (**b**) on the surface of porcine aorta and aortic valve leaflet during a 30-min incubation with 50 μM ATP. Values represent mean ± SEM, *n* = 4; *p < 0.05

**Fig. 2:**
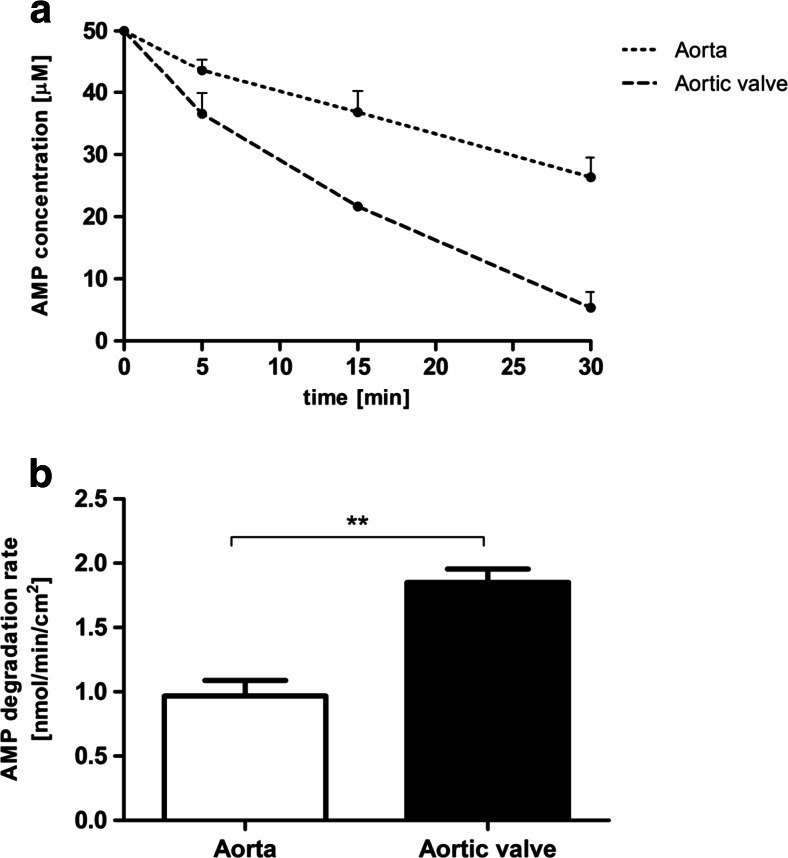
Adenosine monophosphate (AMP) degrading capacity is increased on the aortic valve surface as compared to the vessel surface. AMP concentration during test (**a**) and calculated degradation rate of AMP (**b**) on the surface of porcine aorta and aortic valve leaflet during a 30-min incubation with 50 μM AMP. Values represent mean ± SEM, *n* = 4; **p < 0.01

**Fig. 3:**
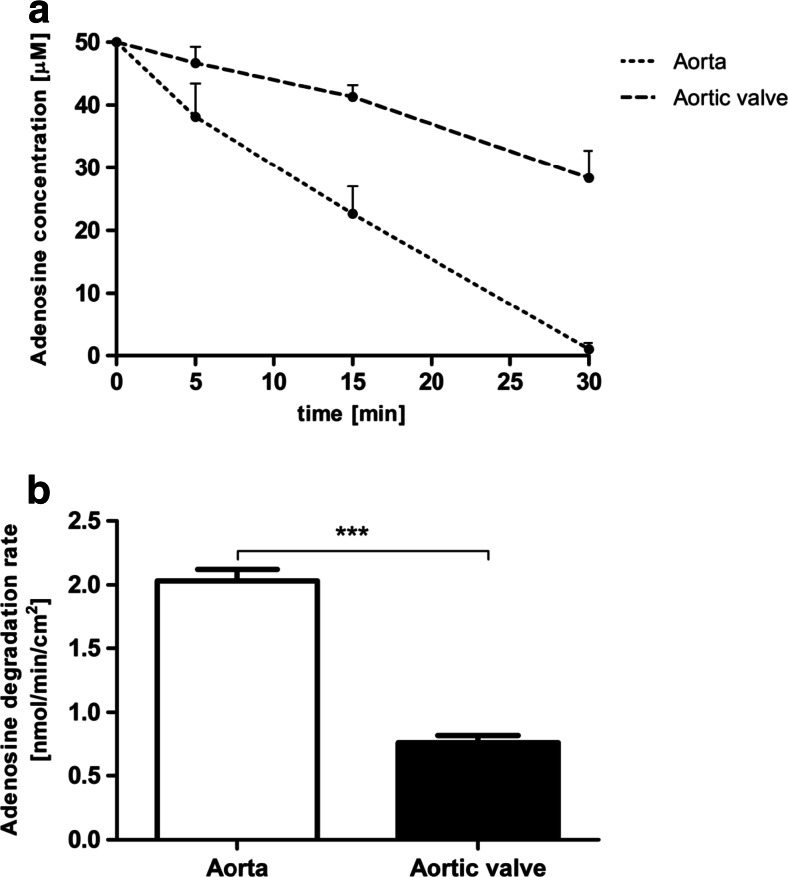
Adenosine degrading capacity is decreased on the aortic valve surface as compared to the vessel surface. Adenosine concentration during test (**a**) and calculated degradation rate of adenosine (**b**) on the surface of porcine aorta and aortic valve leaflet during a 30-min incubation with 50 μM adenosine. Values represent mean ± SEM, *n* = 4; ***p < 0.001

In order to examine the extracellular adenine nucleotide catabolism, the rates of ATP and AMP hydrolysis as well as adenosine degradation were tested on the native valve surface. Intact porcine aortic valve leaflets were characterized by two times higher activities of enzymes engaged in ATP (Fig. 1) and AMP hydrolysis (Fig. 2) in comparison to the aortic wall. Adenosine deaminase activity was approximately 2.5-fold lower on the surface of aortic valve than in aorta (Fig. 3). This demonstrates the special role of extracellular enzymes engaged in nucleotide metabolism in the aortic valve. Observed ecto-enzymes pattern, determined by the fast rate of nucleotides hydrolysis and slowed degradation of adenosine may be beneficial in the preventing of aortic valve inflammation, by the retention of immunomodulatory adenosine.

**Fig. 4:**
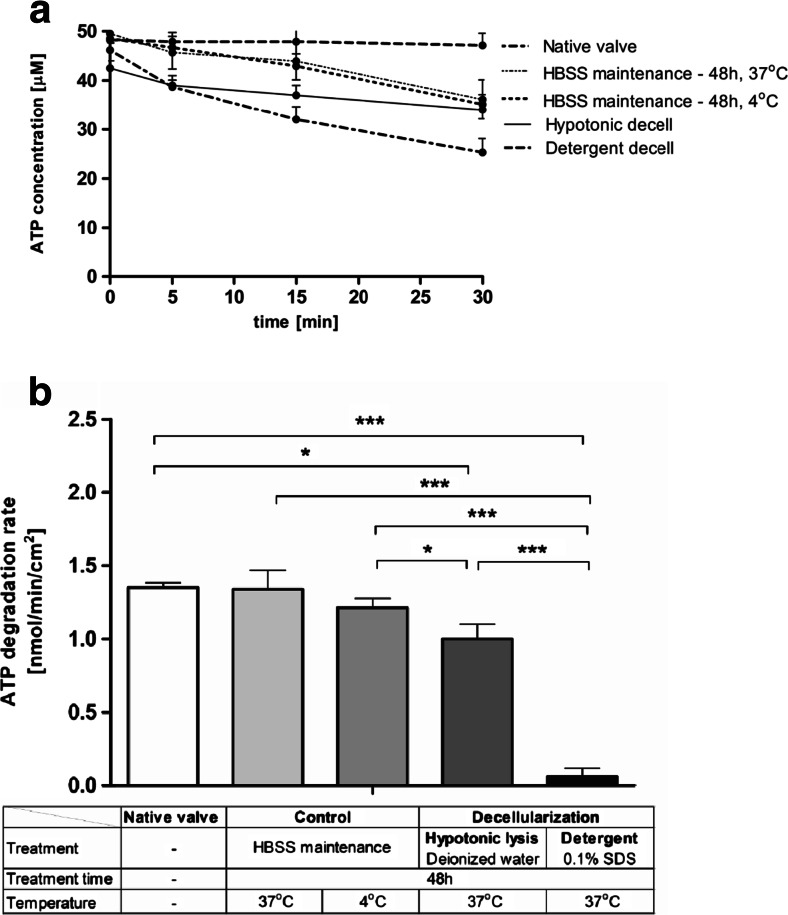
Adenosine triphosphate (ATP) degradation rate is preserved on the surface of the aortic valve processed by hypotonic lysis in contrast to the native valve, while the detergent treatment completely removes this activity. ATP concentration during test (**a**) and calculated degradation rate of ATP (**b**) on the surface of native and processed porcine aortic valve leaflets, during a 30-min incubation of valve leaflets with 50 μM ATP. Values represent mean ± SEM, *n* = 8; *p < 0.05, **p < 0.01, ***p < 0.001

**Fig. 5:**
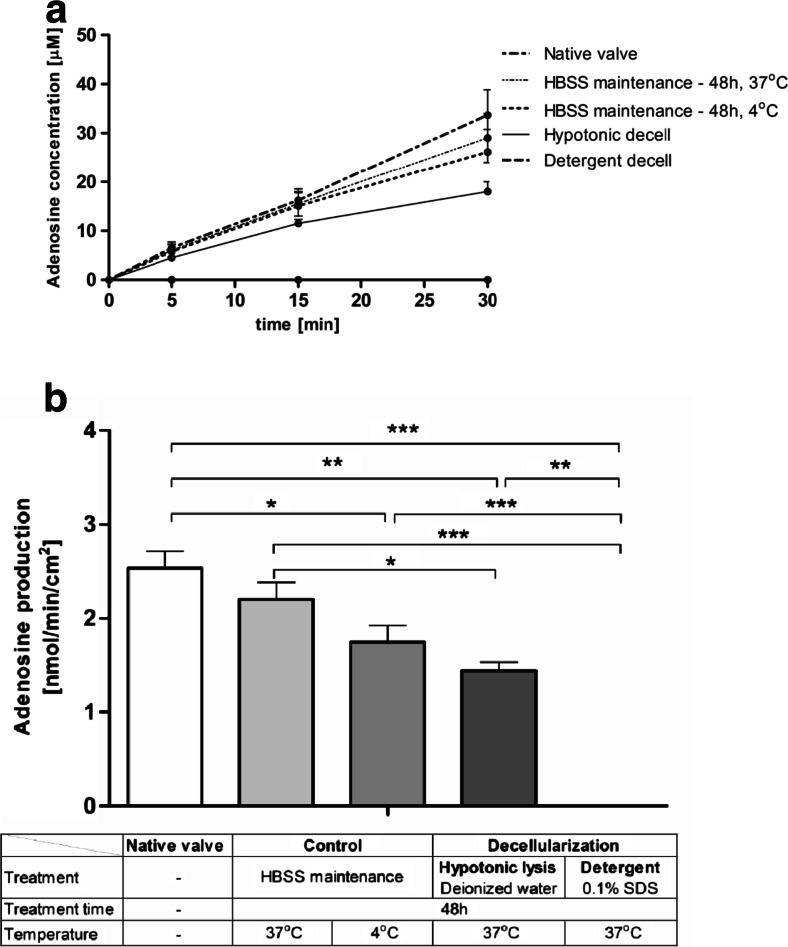
Adenosine production rate is preserved on the surface of the aortic valve processed by hypotonic lysis in contrast to the native valve, while the detergent treatment completely removed this activity. Adenosine concentration during test (**a**) and calculated production rate of adenosine (**b**) on the surface of native and processed porcine aortic valve leaflets, during a 30-min incubation of valve leaflets with 50 μM AMP. Values represent mean ± SEM, *n* = 8; *p < 0.05, **p < 0.01, ***p < 0.001

The next step was to investigate, whether the aortic valve decellularization process affects the activities of extracellular nucleotide metabolism, what is important in terms of valvular prostheses adaptation. Two decellularization techniques were compared. It was revealed that the rate of ATP hydrolysis on the surface of processed valves greatly differed from the native valves (Fig. 4). After decellularization in hypotonic solution, ATP hydrolysis was preserved on the surface of aortic valve leaflets, whereas it was almost completely removed after detergent decellularization. A 48-h incubation in Hank’s solution at 4 and 37 °C only slightly affected the rate of ATP hydrolysis. Similarly, rate of adenosine production (AMP hydrolysis) was completely removed from detergent-decellularized leaflets while it was maintained after hypotonic lysis and 48-h incubation in Hank’s solution (Fig. 5). The increase in the ATP degradation entails a reduction of its proinflammatory properties, what seems to be beneficial in the aortic valve regeneration. On the other hand, only detergent-based decellularization technique seems to solve the problem of remaining on the valve surface enzymes immunogenicity.

**Fig. 6:**
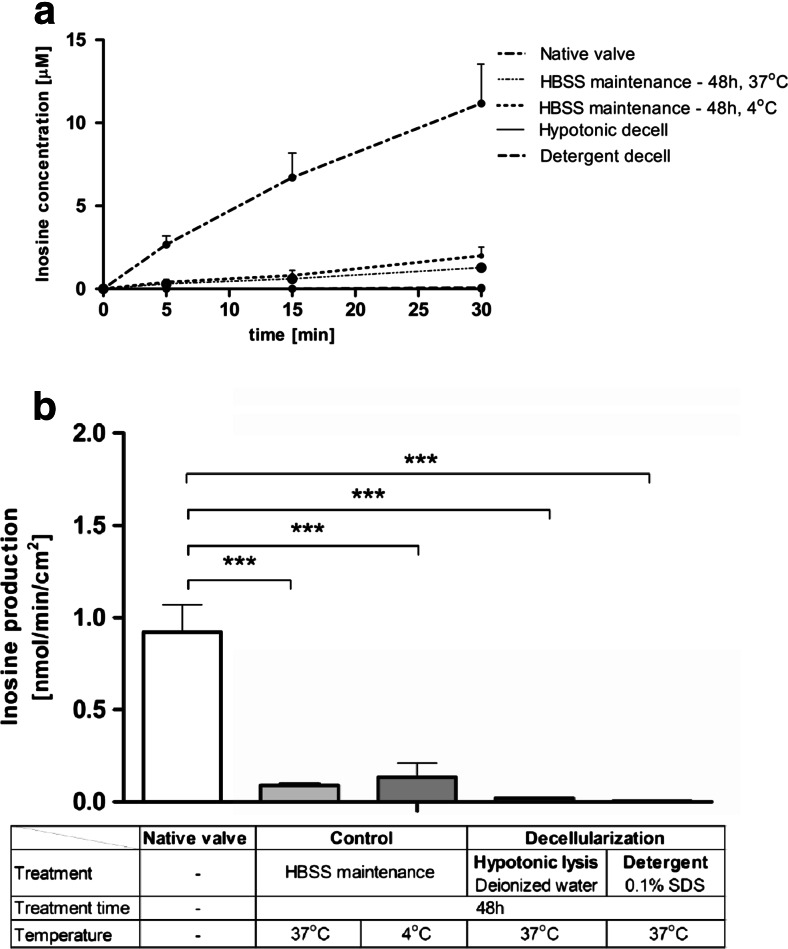
Inosine production rate is profoundly decreased on the surface of the aortic valve after control incubation regarding to the native valve and completely removed after decellularization. Inosine concentration during test (**a**) and calculated production rate (**b**) of inosine on the surface of native and processed porcine aortic valve leaflets, during a 30-min incubation of valve leaflets with 50 μM adenosine. Values represent mean ± SEM, *n* = 8; *p < 0.05, **p < 0.01, ***p < 0.001

Adenosine deaminase activity was six-fold lower after 2 days of incubation, and both methods of decellularization entirely removed its activity (Fig. 6). The reduced adenosine deaminase activity in the control assay (after 2 days of incubation) testifies to the fact that the data obtained from the processed valve surface are not only a result of decellularization. This may be due to the instability of the enzyme activity or unstable adenosine deaminase anchoring in the cell membrane.

Blocking the nucleoside transport by NBTI did not affect the rates of substrate conversion (data not shown) indicating that only extracellular adenosine metabolism took place.

## Discussion

This study supports the concept of special role of extracellular nucleotide metabolism on the surface of heart valves. Activities that convert ATP to AMP and further to adenosine in the valve leaflets far exceeded those found in the aortic wall. Furthermore, we demonstrated diverse effects of procedures for decellularization of xenograft valves for implantation in humans. Some preserved nucleotide degrading activities, while others caused their complete loss. This could have implications for processes directly related to valve longevity. Altered concentrations of nucleotide metabolites will affect their signaling mechanisms and in turn, may affect pathologically significant processes such as thrombosis, inflammation, and calcification and, ultimately, could have important implications for the durability of generated valve bioprostheses.

Many literature data confirm the efficiency of used decellularization techniques, including hypotonic lysis followed by nuclease digestion [15], which is patented as Synergraft® [35]. The effectiveness of the second, detergent-based technique has been repeatedly confirmed histologically by, e.g., H&E staining and Mason’s trichrome, as well as by electron microscopy, which indicated no cell nuclei and cellular components after decellularization protocol, but still preserved essential extracellular matrix proteins, such as collagen and elastin [36]. Our previous results already demonstrated the presence of enzymes activities engaged in extracellular nucleotide metabolism in the aortic valve, which favored the production and retention of extracellular adenosine [29, 37]. Our current data confirmed these alterations in valvular ecto-enzyme activities that could play the important role in valve pathology and its regeneration.

Porcine aortic valves leaflets decellularized by hypotonic lysis followed by nuclease treatment and ɣ-irradiation maintain almost intact activities of nucleotide-degrading ectonucleotidases. One possible mechanism for such effect is retention of cell membranes within decellularized valve scaffold. Preservation of ectonucleotidases may affect processes that are relevant to early and late valve function. Maintenance of ATP degradation and adenosine production capacity could protect the valve against inflammation/calcific degeneration by facilitation of pro-calcific ATP removal and production of protective adenosine [18]. Also, our unpublished data, obtained from the calcified and non-calcified human aortic valves, demonstrate the significant involvement of extracellular nucleotide catabolism in control of the aortic valve pathology. Additionally, literature data suggest protective properties of the extracellular adenosine in the context of implant adaptation. Tuskamoto et al. shown that deficiency of ecto-5′-nucleotidase (CD73) causes enhanced graft-versus-host-disease (GVHD) severity [38]. Both mutant CD73−/− mice, as well as mice with pharmacological blockade of CD73 were characterized by stronger cytotoxicity, cell expansion, and increased proinflammatory cytokine concentration. However, potential disadvantage that is highlighted by retention of enzyme activities is an immune response. Retention of active enzymes indicates retention of immunogenic proteins that constitutes an important risk of graft rejection [39]. Conversely, the use of a detergent-based decellularization protocol completely reduced of all ecto-enzymes activities. While such valves are less likely to be immunogenic, nonviable valves devoid of potentially protective ectonucleotidases could be prone to degeneration and calcification [40]. Optimizing valve preparation technology to maintain or restore ATP degrading ecto-enzymes on the surface of bioprostheses seems to be a promising solution to improve their quality.

The optimal approach could be recellularization of entirely decellularized valves to obtain an implantable bioengineered construct that shows the benefit of healthy valvular tissue that has the potential ability to regenerate or remodel during its lifetime in vivo. The first recellularization concept involves the implantation of decellularized heart valves, which will be reseeded in vivo in the patient’s body. Clinical data from this field revealed successful effects [41, 42]; nevertheless, others have shown a failure of xenogenic scaffolds to reseed [39]. Alternatively, a second approach comprises precise decellularization and elimination of all cellular remnants followed by in vitro cells reseeding. Valvular endothelial cells (VECs) and valvular interstitial cells (VICs) are needed for reseeding. However, since these cells cannot be directly harvested, alternate cell populations are required. Attempts were made to use venous endothelial cell, obtained after surgical procedure [43, 44]. As terminally differentiated cell types, endothelial cells demonstrated many advantages, such as antithrombotic properties that are also provided by effectively functioning extracellular nucleotide catabolism [45, 46]. Moreover, endothelium covers the collagen surface, which could protect against possible immunogenic reactions. This may be caused by cytoprotective effect of immunosuppressive adenosine generated by endothelial ecto-enzymes [47, 48]. However, endothelial cell harvest and expansion in vitro are slow. Endothelial cell reseeding procedures fail due to a weakly adherent endothelium whose survival post implantation is likely very limited. Another approach is to modify the decellularized tissue with molecules that could capture circulating endothelial progenitor cells to the surface of the valve after implantation into the recipient. Moreover, the utilization of adipose-derived cells deserves attention. Adipose-derived stem cells (ADSCs) or stromal vascular fraction cells (AD-SVF) [49] could be successful due to their relatively effective isolation from lipoaspirate obtained through route liposuction procedures and recorded ability to differentiate into various cell lineages [50]. It is documented that ADSCs differentiate into cells expressing markers characteristic for valvular interstitial cells (fibroblast-like cells) [51], but extracellular nucleotide catabolism enzymes specific for these cells needs to be investigated further. It seems that development of new recellularization strategies based on adipose-derived cells, like interstitial seeding techniques by injection of ADSCs directly into the valve leaflet structure, could be promising to obtain a bioprosthesis that closely resembles a native aortic heart valve [50]. The other solution could be the application of the methods used in the production of therapeutic proteins such as genetic engineering including gene isolation or modification which affect the reduction of enzyme immunogenicity and have a favorable effect on the bioprosthesis adaptation [52, 53]. While these strategies are likely to provide ultimate solution for optimal valve graft, optimizing xenograft valve preparation strategy that takes into account nucleotide metabolic mechanisms may provide good intermediate solution.

In conclusion, we have shown that extracellular adenine nucleotide catabolism operates at highest rates on the aortic heart valve surface. Decellularization techniques used in xenograft preparation affects this process in different ways. Some methods preserve capability to degrade ATP to adenosine while others completely remove these activities. We assume that retention of nucleotide degrading capacity protects valves from thrombosis, inflammation and calcification. Therefore, the presence of extracellular nucleotide metabolism enzymes on the valve prostheses is a potential approach for improvement of the valve graft quality. However, this concept requires further studies.

## Abbreviations

ADA: adenosine deaminase
ADSCs: adipose-derived stem cells
AD-SVF: adipose-derived stromal vascular fraction cells
ADP: adenosine diphosphate
AMP: adenosine monophosphate
ATP: adenosine triphosphate
AVR: aortic valve replacement
CAVD: calcific aortic valve disease
DMEM: Dulbecco’s Modified Eagle’s Medium
DMSO: dimethyl sulfoxide
e5’-NT: ecto-5’-nucleotidase
ECM: extracellular matrix
EDTA: ethylenediaminetetraacetic acid
EHNA: erythro-9-(2-hydroxy-3-nonyl) adenine
eNTPD: ecto-nucleoside triphosphate diphosphohydrolase
HBSS: Hank’s balanced salt solution
HPLC: high performance liquid chromatography
NBTI: S-(4-Nitrobenzyl)-6-thioinosine
PBS: phosphate-buffered saline
SDS: sodium dodecyl sulfate
TAVI: transcatheter aortic valve implantation
VECs: valvular endothelial cells
VICs: valvular interstitial cells
